# Embryonal Rhabdomyosarcoma in Mastoid and Middle Ear in a 3-Year-Old Girl: A Rare Case Report

**DOI:** 10.1155/2012/871235

**Published:** 2012-06-18

**Authors:** Saman Vegari, Alireza Hemati, Hosein Baybordi, Leila Davarimajd, Ghasem Chatrbahr

**Affiliations:** ^1^Department of Otolaryngology Head and Neck Surgery, Tabriz Medical Sciences University, Tabriz 5174867773, Iran; ^2^Department of Pathology, Tabriz Medical Sciences University, Tabriz 5174867773, Iran; ^3^Department of Library, Tabriz Medical Sciences University, Tabriz 5174867773, Iran

## Abstract

*Introduction*. The most common sarcoma of childhood is rhabdomyosarcoma, approximately 35% of all paediatric rhabdomyosarcomas occur in the head and neck. *Case Report*. A 3-year-old girl referred to our clinic due to serosanguineous purulent discharge from her right ear. After paraclinical and pathologic evaluation it was diagnosed as embryonic rhabdomyosarcoma. *Conclusions*. In all children with mastoiditis, especially in young children, rhabdomyosarcoma should be considered as a differential diagnosis.

## 1. Introduction

 The most common sarcoma of childhood is rhabdomyosarcoma [1, 2], this tumor is the third most common neoplasm in childhood after neuroblastoma and nephroblastoma [3, 4]. Approximately 35% of all paediatric rhabdomyosarcomas occur in the head and neck [3, 5]. The most common site that involved by rhabdomyosarcoma is orbit (about one-third of cases). After that, in decreasing order, rhabdomyosarcoma affects oral cavity and pharynx (29%), the face and neck region (24%) [3], involvement of the ear and temporal bone with rhabdomyosarcoma is uncommon [6]. Pathologic subtypes of rhabdomyosarcoma include: embryonal, botryoid, alveolar, pleomorphic, spindle cell, and anaplastic variants [7]. The embryonal rhabdomyosarcoma includes about 60–70% of rhabdomyosarcoma cases [8, 9].

## 2. Case Presentation

**Figure 1:**
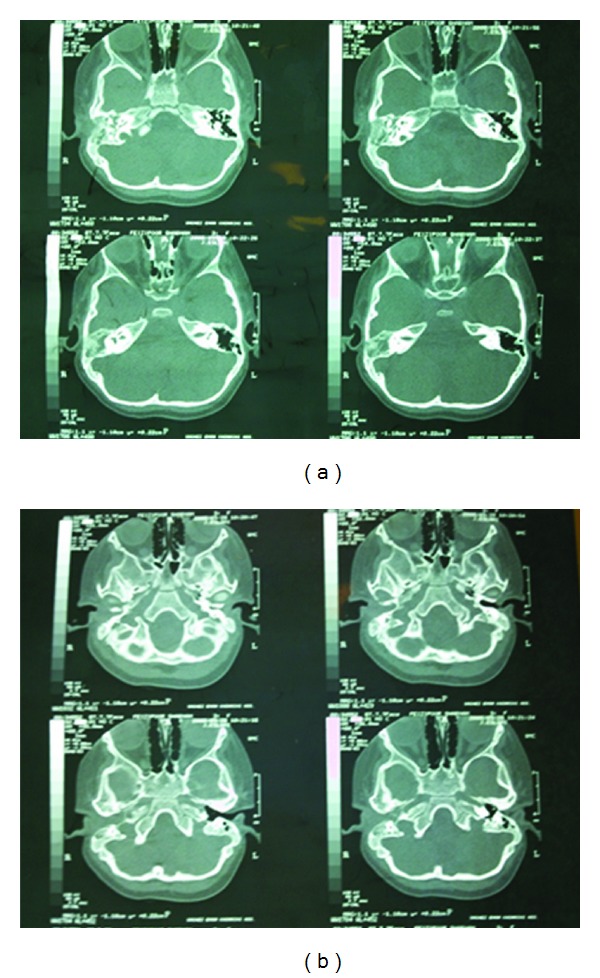
The mass involved external ear and projected from external auditory meatus. There was ragged erosion in mastoid air cells and external surface of mastoid bone.

**Figure 2:**
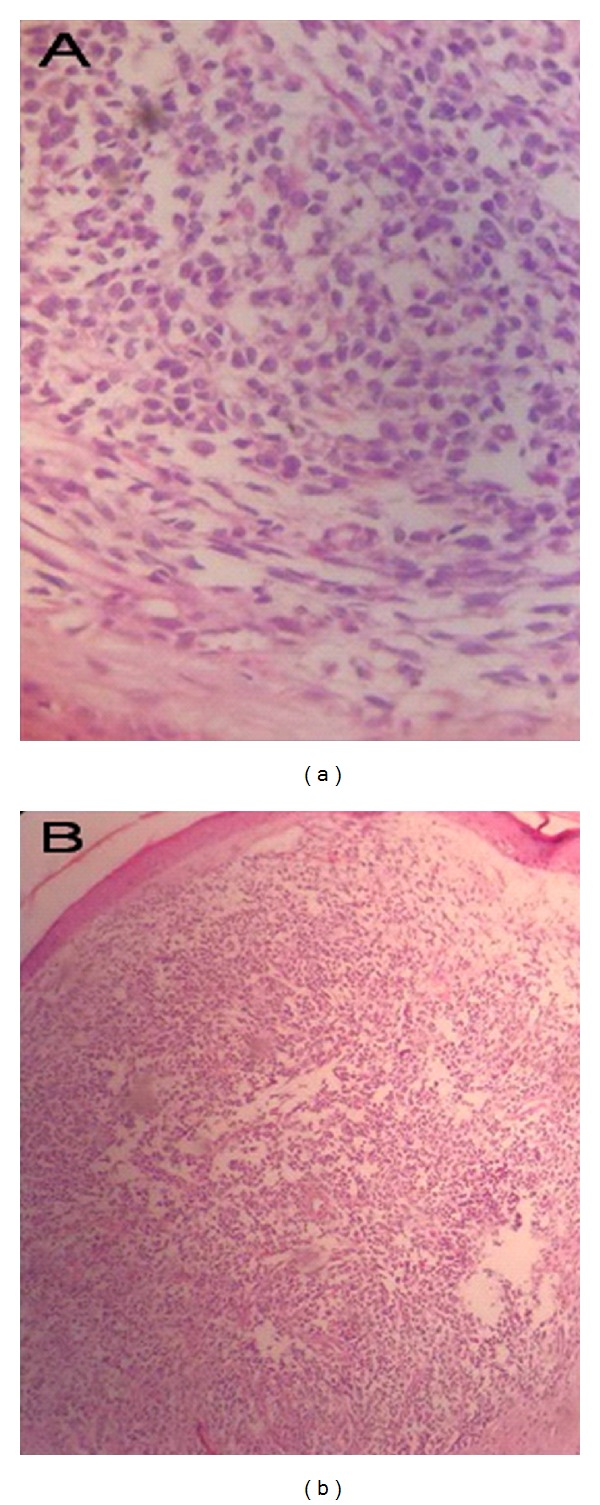
The cells show a combination of shapes but a spindle or elongated morphology is evident overall. Strap cells that look like primitive muscle cells that classically describe in rhabdomyosarcoma are seen in this pictures.

 A 3-year-old girl referred to our clinic due to serosanguineous purulent discharge from her right ear. She was treated with antibiotics for a 3-week period with attenuation in the amount of drainage. In physical examination, there was a polypoid, reddish and fragile mass that filled external ear canal in right side, facial nerve was intact, and there was fetor discharge from the ear canal. Biochemistry tests were normal. In computer tomography, soft tissue density was present in mastoid and middle ear. The mass involved external ear and projected from external auditory meatus. There was ragged erosion in mastoid air cells and external surface of mastoid bone (Figure 1). Therefore, first diagnosis was neoplastic change and rhabdomyosarcoma was most probably because of patient age, metastatic neuroblastoma, lymphoma and leukemia was in differential diagnosis. Patient admitted and biopsy were done from the mass, pathologist reported keratinizing squamous epithelium, granulation tissue and abscess formation. After 2-week radical mastoidectomy was done via postauricular. During surgery external auditory canal and mastoid was full of polypoid granulomatous tissue while tympanic membrane was intact without perforation. Two samples were prepared and sent to two different pathology laboratories, first pathologist reported granulation tissue and keratinizing squamous epithelium, while the second pathologist reported embryonal rhabdomyosarcoma (Figure 2). Then multiagent chemotherapy and radiotherapy were suggested to patient parents and they did not accept to keep on treatment.

## 3. Discussion

Rhabdomyosarcomas may originate in any anatomical site, occurring predominantly in head and neck regions, orbits, skull base, nasal cavity, and nasopharynx, where there is little or no musculoskeletal tissue [2, 10]. In pediatric cases, about 30 to 40% occur in the head and neck regions [3, 5, 10]; the ear and the temporal bone are uncommon sites of involvement. Chao et al. reported 3 patients with temporal bone involvement out of 102 cases [6].

 In 1966 Potter reported a 3-year-old male with bilateral otitis media and polypoid mass in right external ear canal. The patient was diagnosed as having rhabdomyosarcoma [11].

Kukwa et al. in 2011 reported an embryonal rhabdomyosarcoma of the larynx in a 33-year-old man. After unsuccessful chemotherapy, hemilaryngectomy was performed. In follow-up CT no signs of recurrence were found. Recently patient was recurrence free for 62 months [12].

In 2008, Khatami et al. reported a case of congenital rhabdomyosarcoma in one-day-newborn, presented with huge mass in right hand and palpable lymph node in submaxillary [13].

In the 1960s, fewer than one-third of children with rhabdomyosarcoma survived, but cure rates are now approximatly 70%, largely reflecting advances made by the Intergroup Rhabdomyosarcoma Study Committee (IRSC) [3].

## 4. Conclusions

 In all children with mastoiditis, especially in young children, rhabdomyosarcoma should be considered as a differential diagnosis.
